# Serum Creatinine Reference Limits in Pediatric Population—A Single Center Electronic Health Record-Based Database in Taiwan

**DOI:** 10.3389/fped.2021.793446

**Published:** 2021-12-30

**Authors:** Gwo-Tsann Chuang, I-Jung Tsai, Yong-Kwei Tsau

**Affiliations:** Department of Pediatrics, National Taiwan University Children's Hospital, Taipei, Taiwan

**Keywords:** serum creatinine, pediatrics, acute kidney injury, adolescents, renal function

## Abstract

**Objective:** To assess age- and sex-specific serum creatinine levels in a pediatric population using a hospital-based database in Taiwan.

**Study Design:** Data on serum creatinine levels were obtained from the National Taiwan University Hospital-integrated Medical Database (NTUH-iMD). Due to the possibility of having acute kidney injury or chronic kidney disease, individuals with multiple serum creatinine measurements were excluded, and outliers in each age- and sex-specific group were also subsequently removed. The remaining creatinine measurements in each group were analyzed, and 95% reference limits were established.

**Results:** Serum creatinine data of individuals aged between 1 month and 18 years from May 2011 to January 2018 were retrieved. After applying the exclusion criteria, 27,911 individuals with a single corresponding serum creatinine measurement were enrolled. Creatinine level reference limits for each age- and sex-specific group were generated. The upper reference limits (URLs), which are particularly useful in clinical practice, followed the natural trend of increasing serum creatinine with age.

**Conclusion:** We generated serum creatinine reference limits from a single hospital-integrated medical database in Taiwan for different age- and sex-specific groups of children. Our results will aid physicians in clinical practice regarding renal function evaluation, especially for patients without a recent baseline serum creatinine level.

## Introduction

Serum creatinine is an extremely useful index when it comes to assessing renal function (1). Other than glomerular filtration rate (GFR), muscle mass has a strong influence on serum creatinine level (2). From 1 month of age, serum creatinine increases gradually with age in children because of an increase in muscle mass, and consequently the level differs every year. Thus, reference ranges presented in broad age groups (such as 0–4, 4–7, and 7–10 years old) would be insufficient to use clinically for children. For example, the reference cited by the Nelson Textbook of Pediatrics, which most general pediatricians use, states that the serum creatinine URL is 0.50 mg/dL for children under 4 years of age (3, 4). However, using this can lead to underestimation of abnormal renal function in infants and toddlers.

Therefore, it is crucial to establish serum creatinine level reference limits for a particular pediatric population, especially for those who are hospitalized. Once established, creatinine values above the URLs could alert clinicians to further evaluate the patient's renal function to avoid iatrogenic kidney injury, such as monitoring medications and fluid status. Therefore, the aim of this study was to generate age- and sex-specific serum creatinine reference limits using data from a hospital-based database for clinical use among children in Taiwan and even other countries in East Asia.

## Methods

### Study Population

We retrieved serum creatinine data of individuals aged between 1 month and 18 years from May 2011 to January 2018 from the NTUH-iMD. All data were pseudonymized, and thus this retrospective study was exempted from a full ethical review of informed consent by the Institutional Review Board of National Taiwan University Hospital (202102032RIND).

### Serum Creatinine Measurements

Serum creatinine was measured using a chemical analyzer (AU5800, Beckman Coulter, Inc., Brea, California) with the compensated kinetic Jaffe method. Calibration of this creatinine procedure for serum determination was accomplished using a Chemistry Calibrator (Cat # DR0070, Beckman Coulter, Inc.), which is traceable to an isotope dilution mass spectrometry reference method using the National Institutes of Standards and Technology (NIST) Standard Reference Material 967. The within-run precision for serum samples was <3% coefficient of variation CV), and the overall precision was <6% CV.

### Study Groups

Apart from infants, who were defined as those aged from 1 month to 1 year, all other age groups were partitioned in 1-year intervals (e.g., ≥1 year to <2 years of age). All of the children were also analyzed by sex, thus forming 36 age- and sex-specific groups.

### Data Exclusion

Serum creatinine is not as frequently measured in children as in adults, and the majority of pediatric patients who visit our hospital do not have underlying kidney diseases or injuries. Therefore, assuming that the patients requiring serial renal function follow-up were more likely to have underlying renal diseases, or acute kidney injury (AKI) and chronic kidney disease (CKD) as the consequence of infection, heart surgery, congenital heart diseases or critically ill patients after intensive care, etc., we excluded patients who had multiple serum creatinine measurements during the study period.

Some patients with abnormal renal function may have had only one creatinine measurement, if the primary physicians were not aware of the normal creatinine range in children at different age groups. We removed the outliers according to the Tukey method (defined as data points above the third quartile plus 1.5 times the interquartile range) to counter this situation (5).

### Statistical Analysis

Statistical analyses were performed with Microsoft Excel 2016 (Microsoft, Seattle, WA, USA). All of the remaining data were near to a normal population after excluding those mentioned above. For children of the same age, creatinine levels should be distributed normally (6, 7). In the analysis, means, standard deviations (SDs), and percentiles P2.5 and P97.5 were determined. In addition, normally distributed data allowed us to calculate the parametric 97.5 and 2.5th percentile values via mean ±1.96 SD. The P97.5 values of each group were the age- and sex-specific URLs for serum creatinine.

## Results

**Figure 1:**
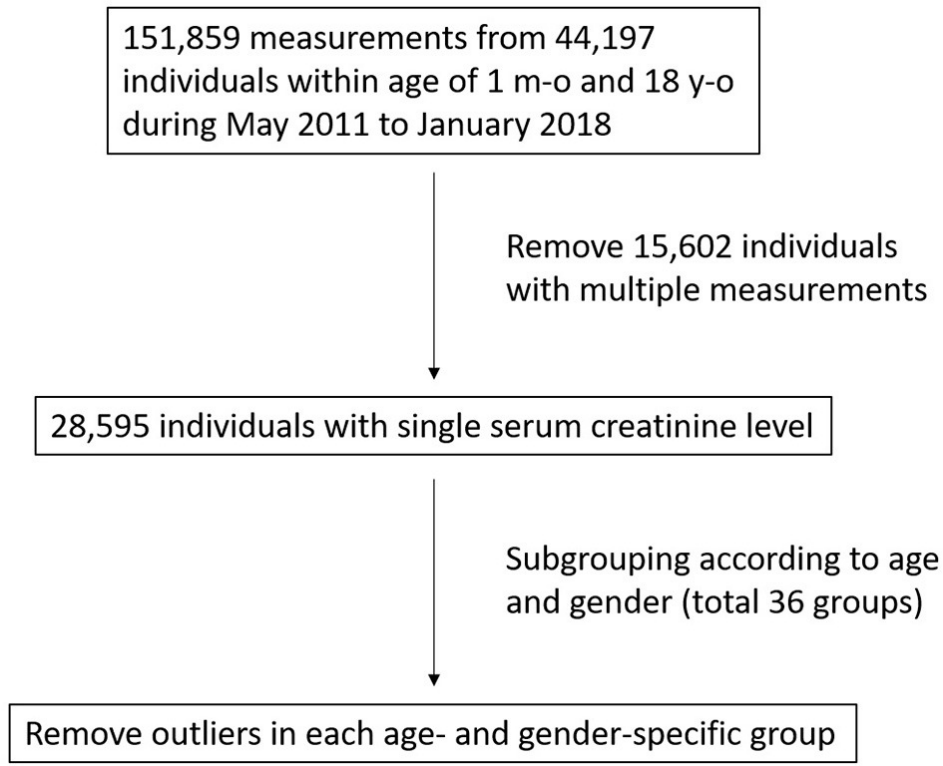
Study population enrollment flowchart.

A total of 151,859 serum creatinine measurements from 44,197 children aged from 1 month to 18 years of age were retrieved (Figure 1). After excluding individuals with multiple measurements, 28,595 remained with a single corresponding serum creatinine measurement. For each of the 36 age-and sex-specific groups, outliers above the third quartile plus 1.5 times the interquartile range were removed. A total of 684 (2.4%) outliers were removed, and 27,911 individuals (15,391 males and 12,520 females) remained for subsequent analysis. The mean, 2.5 and 97.5th percentiles of serum creatinine in each group are shown in Table 1. There was a trend that the serum creatinine level increased with age. In the adolescent period (>13 years of age), the URLs of serum creatinine in the males were higher than in the females, with differences ranging from 0.11 to 0.23 mg/dL.

**Table 1:** Reference intervals for serum creatinine (mg/dL) in each age- and sex-specific group.

**Groups**		**Number of individuals**	**Mean ± SD**	**P2.5**	**P97.5**
**Age**	**Sex**				
Infant (1–12 months)	M	2,227	0.26 ± 0.07	0.12	0.39
	F	1,527	0.25 ± 0.07	0.11	0.39
1–2 years	M	1,653	0.29 ± 0.07	0.15	0.42
	F	1,367	0.29 ± 0.07	0.15	0.42
2–3 years	M	1,230	0.33 ± 0.07	0.19	0.46
	F	1,012	0.31 ± 0.07	0.17	0.45
3–4 years	M	1,011	0.35 ± 0.07	0.21	0.49
	F	824	0.34 ± 0.07	0.20	0.48
4–5 years	M	946	0.37 ± 0.07	0.23	0.51
	F	708	0.37 ± 0.07	0.23	0.50
5–6 years	M	842	0.39 ± 0.07	0.25	0.53
	F	662	0.38 ± 0.07	0.24	0.52
6–7 years	M	723	0.42 ± 0.08	0.26	0.57
	F	565	0.41 ± 0.08	0.25	0.56
7–8 years	M	602	0.44 ± 0.08	0.28	0.59
	F	495	0.44 ± 0.08	0.28	0.60
8–9 years	M	559	0.46 ± 0.08	0.30	0.61
	F	454	0.46 ± 0.08	0.30	0.61
9–10 years	M	519	0.49 ± 0.08	0.33	0.63
	F	452	0.47 ± 0.08	0.31	0.62
10–11 years	M	491	0.52 ± 0.08	0.36	0.68
	F	371	0.49 ± 0.07	0.35	0.63
11–12 years	M	496	0.54 ± 0.09	0.36	0.71
	F	381	0.51 ± 0.09	0.33	0.70
12–13 years	M	517	0.58 ± 0.10	0.38	0.78
	F	399	0.54 ± 0.09	0.36	0.73
13–14 years	M	487	0.64 ± 0.11	0.42	0.85
	F	397	0.58 ± 0.08	0.42	0.74
14–15 years	M	465	0.72 ± 0.11	0.50	0.94
	F	419	0.61 ± 0.09	0.43	0.79
15–16 years	M	619	0.79 ± 0.12	0.55	1.02
	F	550	0.62 ± 0.10	0.42	0.81
16–17 years	M	726	0.82 ± 0.12	0.58	1.05
	F	708	0.63 ± 0.09	0.45	0.82
17–18 years	M	1,278	0.84 ± 0.11	0.62	1.05
	F	1,229	0.64 ± 0.09	0.46	0.82

To confirm that few patients with AKI or CKD remained after the exclusion steps, we also reviewed the diagnosis codes of each individual in the final analysis stage, and the percentage of patients with any kidney or urinary tract diseases was 2.1% (596/27,911). For each subgroup, the same mean and SD of serum creatinine value after rounding up to two decimal places were obtained regardless of whether or not these individuals were included (Supplementary Table S1).

## Discussion

In this study, we generated age- and sex-specific serum creatinine URLs for children using data from our hospital's laboratory information system. Neonates were excluded from the analysis, as the initial serum creatinine peak after birth is greatly determined by maternal serum creatinine, and the level then declines persistently throughout the first 4 weeks of life (8). Consequently, a reference for this age group would not be consistent and thus not informative. Applying pediatric estimated GFR (eGFR) formulas for neonates is also inappropriate, and it is more important to compare current creatinine levels to previous data during this period (9).

We compared our results with three other studies (10–12) that also addressed pediatric age- and sex-specific serum creatinine URLs (Table 2). These studies were selected for comparison since they also used narrow age-range groups to assess pediatric age- and sex-specific serum creatinine reference limits in contrast to the many existing studies on broad age-range groups. Serum creatinine URLs in studies by Pottel et al. (10) and Uemura et al. (11) had several reversion points, violating the rule that serum creatinine levels normally increases with age; thus, it is unclear whether these URLs could accurately reflect a normal population. The presence of reversion points may be due to the small sample size in each adolescent group in their studies, as many groups included fewer than 120 individuals, which is below the minimum number required to establish reference values recommended by the International Federation of Clinical Chemistry and Laboratory Medicine (IFCC) (13). The minimum sample size of a single group was 371 (females aged 10–11 years) in our study, and thus the numbers of subjects in all age- and sex-specific groups were far beyond the IFCC recommendations.

**Table 2:** Upper reference limits (P97.5) of serum creatinine (mg/dL) in different studies.

**Study**	**Pottel**		**Uemura**		**Søeby**		**Current study**	
**Age**	**M**	**F**	**M**	**F**	**M**	**F**	**M**	**F**
Infant (1–12 months)	0.36				0.31		0.39	0.39
1–2 years	0.39		0.32		0.33		0.42	0.42
2–3 years	0.42		0.37		0.38		0.46	0.45
3–4 years	0.46		0.37		0.42		0.49	0.48
4–5 years	0.50		0.40		0.45		0.51	0.50
5–6 years	0.53		0.45		0.51		0.53	0.52
6–7 years	0.58		0.48		0.53		0.57	0.56
7–8 years	0.60		0.49		0.59		0.59	0.60
8–9 years	0.62		0.53		0.60		0.61	0.61
9–10 years	0.69		0.50^a^		0.63		0.63	0.62
10–11 years	0.71		0.57		0.67		0.68	0.63
11–12 years	0.71		0.58		0.70		0.71	0.70
12–13 years	0.74		0.61	0.66	0.72		0.78	0.73
13–14 years	0.83^b^		0.80	0.69	0.79^b^	0.79^b^	0.85	0.74
14–15 years	0.91	0.78^a^	0.96	0.71	0.97	0.85	0.94	0.79
15–16 years	1.01	0.92	0.93^a^	0.72	1.06	0.88	1.02	0.81
16–17 years	1.07	0.95	0.96	0.74	1.11	0.90	1.05	0.82
17–18 years	1.10	0.94^a^			1.12	0.90	1.05	0.82

a *Without following the natural trend of increasing serum creatinine with age*.

b *Same upper reference limits for boys and girls*.

Boys have a faster rate of muscle mass growth due to a greater androgenic effect, and thus the increase in their serum creatinine level is more pronounced than that in girls during adolescence (14). However, Søeby et al. found no significant difference between boys and girls aged 13–14 years (12). Currently, there is no evidence supporting that the peak growth period in Caucasian male adolescents is later than that in East Asian male adolescents. The reasonable explanation is that genetic and environmental factors that might influence muscle mass increments differ between adolescent girls in different races, which eventually caused differences in serum creatinine levels. Søeby et al. concluded that in certain groups, such as 13–14-year-old males and females, the results may be imprecise (12). In addition, Pottel et al. (10) suggested that the reversion point in females aged 14–15 years can also be explained by pooling both boys and girls in the same group at 13–14 years, which is a period when creatinine levels should already be different between males and females.

All three of the studies we used for comparison were also hospital-based. Pottel et al. (8) and Søeby et al. (10) did not review individual diagnoses, and suggested that the prevalence of pediatric renal diseases is low and thus could be neglected. However, the prevalence of children with renal diseases and decreased GFR in a hospital-based study would definitely be higher than in the general population, and would even be expected to vary between local hospitals and tertiary medical centers. In the present study, we reviewed the proportion of patients with any diagnosis relevant to kidney and urinary tract diseases in the final stage, and the rate was very low (only 2.1%). In further analysis, we found that the serum creatinine reference intervals were not altered regardless of whether or not these individuals were included. This also demonstrated that our results were more reliable than the other studies.

The aim of this study was to establish serum creatinine URLs for different sex and age groups, which could then be used by clinicians taking care of hospitalized children. Children with creatinine levels beyond the limits should then undergo further renal function assessments. No equation is currently available to estimate unstable renal function in children, and thus assessing AKI is mostly determined according to the serum creatinine criteria of the “Kidney Disease: Improving Global Outcomes” guidelines (15). A study from a medical center in Taiwan reported that 2.6% of children with hospital admissions had AKI (16). Due to a lack of baseline creatinine level, the incidence of AKI may be largely underestimated, as McGregor et al. reported that least 5% of non-critically ill hospitalized children had one or more AKI episodes during admission (17). As a surrogate, by understanding serum creatinine URL according to the patients' age, early awareness of kidney impairment in certain patients may alert the primary physicians and subsequently adjust the initial patient management that might prevent worsening of renal function during hospitalization.

The strengths of this study include the large sample size, which permits a more accurate estimation of reference values. Although our sample was obtained from a hospital-based retrospective database analysis, the large sample size (18) could also provide the best reference values for use in general practice to help prevent iatrogenic kidney injury in patients during hospitalization. We also reviewed whether individuals with kidney or urinary tract diseases interfered with the results, and thus our results are credible.

The major limitation of this study is that the data were from a medical center rather from the general population. However, it is difficult to obtain blood samples from a large number of healthy children. All hospital-based studies have to confront this problem and generate results via reasonable assumptions and methods. It is also worth mentioning that having a normal creatinine level does not guarantee having a normal GFR all the time. This is true especially among severely malnourished children or those with muscle-wasting diseases.GFR estimation based on serum creatinine and body height may also be imprecise in these children (19, 20). In these situations, measuring creatinine clearance or using cystatin C-based eGFR equations may be a better approach to assess renal function (21).

In conclusion, we generated serum creatinine URLs from a retrospective database analysis of a hospital-based pediatric population in Taiwan. The results are compatible with the natural trend of an increase in serum creatinine with age in children and adolescents. We analyzed boys and girls separately, and differences between adolescent boys and girls were clearly demonstrated. Our results provide information on monitoring renal function in hospitalized children that will help primary physicians in clinical practice to avoid possible iatrogenic renal injury and CKD comorbidities in the future.

## Data Availability Statement

The original contributions presented in the study are included in the article/Supplementary Material, further inquiries can be directed to the corresponding author/s.

## Ethics Statement

The studies involving human participants were reviewed and approved by the Institutional Review Board of National Taiwan University Hospital (202102032RIND). Written informed consent from the participants' legal guardian/next of kin was not required to participate in this study in accordance with the national legislation and the institutional requirements.

## Author Contributions

G-TC and I-JT: conceived and designed the study and analyzed and interpreted the data. G-TC: acquired the data and drafted the manuscript. I-JT and Y-KT: edited the manuscript. G-TC, I-JT, and Y-KT: approved the final version of the manuscript. All authors contributed to the article and approved the submitted version.

## Conflict of Interest

The authors declare that the research was conducted in the absence of any commercial or financial relationships that could be construed as a potential conflict of interest.

## Publisher's Note

All claims expressed in this article are solely those of the authors and do not necessarily represent those of their affiliated organizations, or those of the publisher, the editors and the reviewers. Any product that may be evaluated in this article, or claim that may be made by its manufacturer, is not guaranteed or endorsed by the publisher.
