# Supplementary material for: Serum Creatinine Reference Limits in Pediatric Population—A Single Center Electronic Health Record-Based Database in Taiwan
Source: Front Pediatr. 2021 Dec 30;9:793446. doi: 10.3389/fped.2021.793446 (PMC8756578; doi:10.3389/fped.2021.793446)
Supplement: Supplementary file 1 [file Table_1.pdf]

Supplementary Table S1. Reference intervals for serum creatinine (mg/dL) in each age- and sex-specific group. (Patients with kidney or urinary tract diseases were excluded.)

Groups		Number of individuals	Mean ± SD	P2.5	P97.5
Age	Sex				
Infant (1-12 months)	M	2117	0.26 ± 0.07	0.12	0.39
	F	1439	0.25 ± 0.07	0.11	0.39
1-2 years	M	1638	0.29 ± 0.07	0.15	0.42
	F	1351	0.29 ± 0.07	0.15	0.42
2-3 years	M	1222	0.33 ± 0.07	0.19	0.46
	F	995	0.31 ± 0.07	0.17	0.45
3-4 years	M	998	0.35 ± 0.07	0.21	0.49
	F	812	0.34 ± 0.07	0.20	0.48
4-5 years	M	935	0.37 ± 0.07	0.23	0.51
	F	698	0.37 ± 0.07	0.23	0.5
5-6 years	M	835	0.39 ± 0.07	0.25	0.53
	F	654	0.38 ± 0.07	0.24	0.52
6-7 years	M	712	0.42 ± 0.08	0.26	0.57
	F	547	0.41 ± 0.08	0.25	0.56
7-8 years	M	587	0.44 ± 0.08	0.28	0.59
	F	479	0.44 ± 0.08	0.28	0.6
8-9 years	M	549	0.46 ± 0.08	0.30	0.61
	F	447	0.46 ± 0.08	0.30	0.61
9-10 years	M	503	0.49 ± 0.08	0.33	0.63
	F	440	0.47 ± 0.08	0.31	0.62
10-11 years	M	481	0.52 ± 0.08	0.36	0.68
	F	359	0.49 ± 0.07	0.35	0.63
11-12 years	M	489	0.54 ± 0.09	0.36	0.71
	F	371	0.51 ± 0.09	0.33	0.7
12-13 years	M	503	0.58 ± 0.10	0.38	0.78
	F	395	0.54 ± 0.09	0.36	0.73
13-14 years	M	478	0.64 ± 0.11	0.42	0.85
	F	386	0.58 ± 0.08	0.42	0.74
14-15 years	M	457	0.72 ± 0.11	0.50	0.94
	F	411	0.61 ± 0.09	0.43	0.79
15-16 years	M	609	0.79 ± 0.12	0.55	1.02
	F	541	0.62 ± 0.10	0.42	0.81
16-17 years	M	713	0.82 ± 0.12	0.58	1.05
	F	691	0.63 ± 0.09	0.45	0.82
17-18 years	M	1266	0.84 ± 0.11	0.62	1.05
	F	1207	0.64 ± 0.09	0.46	0.82
